# RAGE inhibits human respiratory syncytial virus syncytium formation by interfering with F-protein function

**DOI:** 10.1099/vir.0.049254-0

**Published:** 2013-08

**Authors:** Jane Tian, Kelly Huang, Subramaniam Krishnan, Catherine Svabek, Daniel C. Rowe, Yambasu Brewah, Miguel Sanjuan, Andriani C. Patera, Roland Kolbeck, Ronald Herbst, Gary P. Sims

**Affiliations:** Research Department, MedImmune LLC, One MedImmune Way, Gaithersburg, MD 20878, USA

## Abstract

Human respiratory syncytial virus (RSV) is a major cause of severe lower respiratory tract infection. Infection is critically dependent on the RSV fusion (F) protein, which mediates fusion between the viral envelope and airway epithelial cells. The F protein is also expressed on infected cells and is responsible for fusion of infected cells with adjacent cells, resulting in the formation of multinucleate syncytia. The receptor for advanced glycation end products (RAGE) is a pattern-recognition receptor that is constitutively highly expressed by type I alveolar epithelial cells. Here, we report that RAGE protected HEK cells from RSV-induced cell death and reduced viral titres *in vitro*. RAGE appeared to interact directly with the F protein, but, rather than inhibiting RSV entry into host cells, virus replication and budding, membrane-expressed RAGE or soluble RAGE blocked F-protein-mediated syncytium formation and sloughing. These data indicate that RAGE may contribute to protecting the lower airways from RSV by inhibiting the formation of syncytia, viral spread, epithelial damage and airway obstruction.

## Introduction

Human respiratory syncytial virus (RSV) is a major cause of severe lower respiratory tract infection in premature and high-risk infants, immunocompromised patients and the elderly (Collins & Melero, 2011). Premature infants and infants with chronic lung or congenital heart diseases are considered high-risk groups for the development of an RSV infection requiring hospitalization (Meissner *et al.*, 2004). Effective RSV vaccines are not available, but a humanized anti-RSV mAb, palivizumab, has been approved for use in the USA since 1998 (Johnson *et al.*, 1997). The non-specific antiviral guanosine analogue ribavirin has been used to treat RSV infections; however, concerns raised over its side-effects and efficacy highlight the need for alternate therapeutics (Leyssen *et al.*, 2008; Sidwell & Barnard, 2006).

RSV is a member of the family *Paramyxoviridae* comprising negative-strand RNA viruses. It is composed of a host-membrane-derived envelope that must attach and fuse with the plasma membrane of the host cell to deliver the viral genetic material. The RSV-encoded attachment (G) protein, fusion (F) protein and small hydrophobic (SH) protein are located in the RSV envelope. The F protein appears to be the most important viral envelope protein for viral infection, as it is capable of mediating attachment and fusion in the absence of the G and SH proteins (Kahn *et al.*, 1999; Techaarpornkul *et al.*, 2001). Upon attachment, RSV F protein is activated by currently unidentified mechanism(s). The activation triggers dramatic conformation changes in the F protein, which allow the fusion peptide of the F protein to be inserted into the plasma membrane of a target cell, resulting in membrane merger and stable fusion pore formation (Melikyan *et al.*, 2000; Russell *et al.*, 2001; Yin *et al.*, 2005, 2006). RSV F protein is also expressed on the plasma membrane of infected cells and mediates cell-to-cell fusion, resulting in multinucleate syncytium formation (König *et al.*, 2004; Morton *et al.*, 2003). Efficient RSV infection appears to involve cell-surface glycosaminoglycans (Hallak *et al.*, 2000), and it was reported recently that nucleolin may act as a receptor for RSV (Tayyari *et al.*, 2011); however, the host receptor(s) that mediate the cell-to-cell fusion and the formation of syncytia remain unknown. Syncytia are found in autopsy samples from fatal RSV infections, but the interstitial inflammation and airway obstruction from epithelial sloughing are the most prominent histopathological features (Johnson *et al.*, 2007; Neilson & Yunis, 1990).

Aspects of RSV infection have been studied extensively in in-bred mice with the use of targeted gene deficiencies. However, RSV is not a natural pathogen of mice and they are relatively resistant to human strains of RSV (Bem *et al.*, 2011). The course of infection and the host response differ from humans in several respects. High titres of virus are necessary to infect mice, the tissue pathology is mild, and the location and composition of the inflammatory cell infiltrate differ significantly. RSV infection of premature lambs more closely resembles the infection of premature infants, with a similar inflammatory response with some histological evidence of epithelial hyperplasia and syncytia, although the actual symptoms of lower respiratory tract infection are minimal (Meyerholz *et al.*, 2004; Olivier *et al.*, 2009; Sow *et al.*, 2011). Infection of cattle with bovine RSV, rather than human RSV, may cause a significant lower respiratory infection but this is often associated with bacterial superinfections, which are extremely uncommon in human infants with RSV infection. Recently, RSV A2 infection of infant baboons was shown to closely parallel the clinical changes, including tachypnea and hypoxia, and pathological features in human infants (Papin *et al.*, 2013). RSV antigen was detected in bronchial epithelium and the alveolar interstitium. The infection appeared to originate in the ciliated bronchial epithelial cells and then spread to the interstitium over the first few days of infection. RSV infection caused fusion of epithelial cells into characteristic syncytia, sloughing of the bronchial epithelium and, in combination with inflammatory infiltrates, obstruction of the bronchioles (Papin *et al.*, 2013).

*In vitro*, RSV infections of well-differentiated bronchial epithelial cultures have not necessarily recapitulated *in vivo* observations. Zhang *et al.* (2002) examined the RSV infection of adult bronchial cells and reported that RSV infection was restricted to the ciliated bronchial columnar epithelial cells and that the virus failed to induce any significant cytopathology over a 3 month period. The authors indicated that the host immune response must drive the tissue pathology *in vivo*. In contrast, a similar study reported complete inhibition of ciliary function in 2 h, syncytia formation from 24 h, epithelial sloughing, and complete depletion of ciliated cells by 3–5 days (Tristram *et al.*, 1998). More recently, RSV infection of well-differentiated paediatric bronchial epithelial cultures also caused syncytia formation and apical cell sloughing (Villenave *et al*., 2012). Considering these *in vitro* data and the differential consequences of RSV infection between healthy adults and susceptible groups, factors besides the host immune response are likely to influence the tissue pathology of RSV infection.

The receptor for advanced glycation end products (RAGE) is a type I transmembrane pattern-recognition receptor that binds a diverse set of endogenous ligands and regulates an array of inflammatory processes (Sims *et al.*, 2010). RAGE is expressed at low levels on multiple cell types and is increased in the bronchial epithelium, airway smooth muscle and alveolar macrophages of smokers and chronic obstructive pulmonary disease patients, and is expressed constitutively at very high levels by type I pneumocysts (Buckley & Ehrhardt, 2010; Ferhani *et al.*, 2010; Shirasawa *et al.*, 2004). Proteolytic cleavage proximal to the membrane and splice variants can generate soluble RAGE which can act as a decoy receptor (Raucci *et al.*, 2008; Yamakawa *et al.*, 2011). Soluble RAGE is released into the airway lumen during RSV infection and lung injury (Buckley & Ehrhardt, 2010; Miller *et al.*, 2012; Uchida *et al.*, 2006).

As RAGE is expressed at very high levels in the lower airways and has been demonstrated to mediate a variety of protective and pro-inflammatory cellular responses (Sims *et al.*, 2010), we tested the hypothesis that RAGE may influence RSV infection. Initial experiments indicated that RAGE had a profound effect on RSV-infected cells, and a series of *in vitro* experiments described here examined the role of RAGE at different stages of RSV infection.

## Results and Discussion

**Fig. 1.**
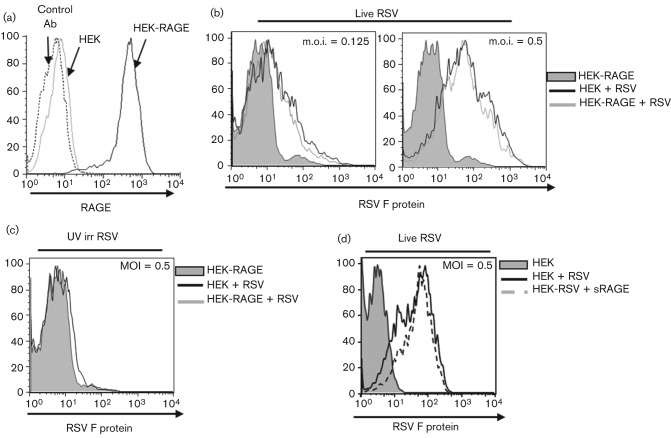
RAGE fails to block F protein-mediated RSV infection. (a) Expression of RAGE on parental HEK and HEK-RAGE cells. Ab, anti-RAGE antibody. (b–d) RSV infection of HEK and HEK-RAGE cells assessed by measuring membrane expression of the F protein by flow cytometry after 16 h. (b) HEK-RAGE cells without viral infection (grey shading), and HEK-RAGE (grey line) or HEK (black line) cells infected with live virus at the indicated m.o.i. (c) HEK-RAGE cells without viral infection (grey shading), and HEK-RAGE (grey line) or HEK (black line) cells infected with UV-irradiated (UV irr) virus at an m.o.i. of 0.5. (d) Parental HEK cells without virus (grey shading), and HEK cells infected with live RSV virus (black line) or with live virus and 300 nM soluble RAGE-Fc (sRAGE) (dotted line).

Understanding the role of RAGE in the lung has been hindered because primary alveolar pneumocytes and epithelial cell lines are either difficult to culture or fail to express physiological levels of RAGE *in vitro* (Shirasawa *et al.*, 2004). To investigate a potential role for RAGE in human RSV infection, we therefore used human HEK293 cells, which are amenable to transfection and susceptible to RSV infection and syncytium formation. Full-length RAGE was stably transfected into HEK293 cells (HEK-RAGE), and surface expression was confirmed with an anti-RAGE antibody (Fig. 1a). We initially determined whether RAGE interfered with the attachment and entry of RSV A2. Shortly after RSV infection, F protein is produced and expressed on the cell surface, and F-protein-specific antibodies can be used to detect infected cells (Huang *et al.*, 2010a). At 16 h post-infection (p.i.), the level of F-protein expression increased in proportion to the m.o.i. of live virus (Fig. 1b). No F protein was expressed on cells challenged with UV-irradiated virus (Fig. 1c), demonstrating that infection by live viral particles is necessary for the cell-surface expression of the F protein. However, there was no difference in the levels of F-protein expression between the HEK and HEK-RAGE cells following RSV infection (Fig. 1b), and pre-treating HEK cells with soluble RAGE before infection also failed to alter F-protein expression (Fig. 1d). These data indicated that RAGE does not impact on the initial attachment and infection of cells by RSV, or the capacity of the infected cell to generate F protein.

**Fig. 2.**
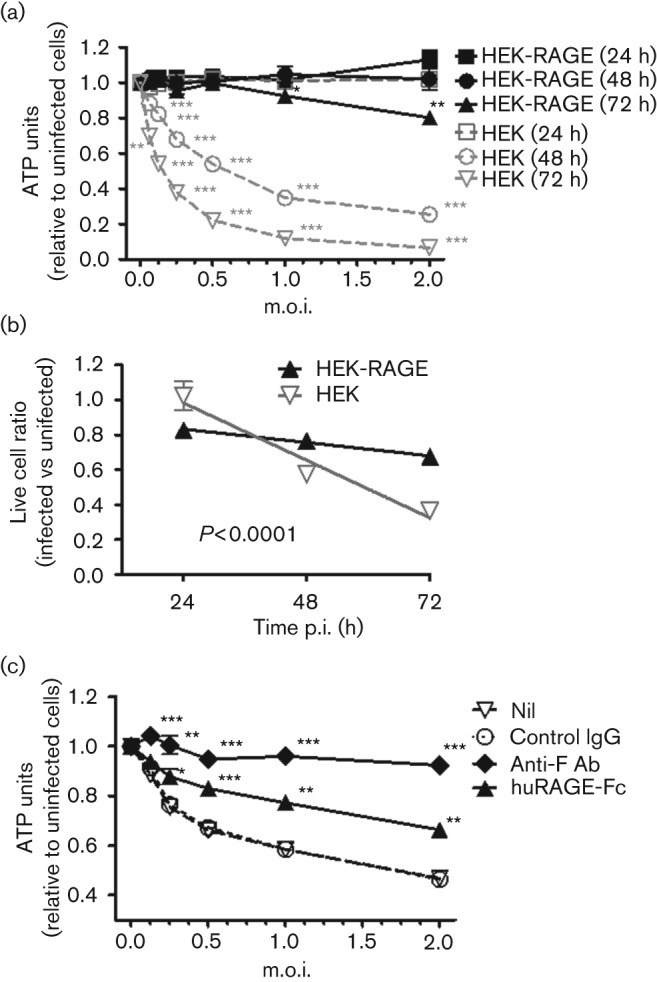
RAGE protects cells from RSV-induced cell death. (a) Parental HEK or HEK-RAGE cells were seeded at 1×10^4^ cells in a 96-well culture plate and incubated alone or with an increasing dose of virus ranging from an m.o.i. of 0.06250 to 2 (i.e. 625 to 2×10^4^ viral particles). ATP units were expressed relative to the uninfected cell cultures for HEK cells (grey symbols) and HEK-RAGE cells (black symbols) at 24 h (squares), 48 h (circles) and 72 h (triangles). Parametric *t*-tests were used to determine the m.o.i. at which RSV significantly impacted the viability of HEK and HEK-RAGE cells. **P<0.01; *** *P*<0.001. (b) Live cell counts for RSV-infected (m.o.i. = 1) HEK cells (grey inverted triangle) and HEK-RAGE (black triangle) after 24, 48 and 72 h. Live cell counts were expressed relative to uninfected cells at the given time point. Curves of lines were generated by linear regression, and the slopes were significantly different (*P*<0.0001). (c) HEK cells were untreated (open triangle) or pre-treated with control IgG (open circle), anti-F protein antibody (Ab) (black diamond) or soluble RAGE-Fc (black triangle) and infected with RSV at the indicated m.o.i., and ATP levels were measured at 48 h. ATP units were expressed relative to uninfected HEK cells. Parametric *t*-tests were used to determine whether the treatment significantly improved the viability of HEKs when compared with the controls. *P<0.05; **P<0.01; ***P<0.001.

However, after 24 h in culture with live RSV A2 virus, it was readily apparent that the HEK-RAGE cells had a clear survival advantage over the parental HEK cells. Cell viability was assessed by total ATP levels over a range of viral doses at 24, 48 and 72 h p.i. (Fig. 2a). ATP levels of the HEK and HEK-RAGE cells were comparable to those of uninfected cells after 24 h. By 48 h, there was a clear dose-dependent reduction in ATP levels in the HEK cell culture, and at 72 h p.i. with an m.o.i. of 1 or more, there was at least an 80 % reduction in ATP levels, indicating that most cells were dying or dead. In contrast, the ATP levels of the HEK-RAGE cells were not significantly affected at 48 h, and a notable decrease was only detected at the highest m.o.i. at 72 h (Fig. 2a). Viable cell counts recovered from the cultures also demonstrate that RAGE significantly protected the HEK cells from RSV-induced cell death (Fig. 2b). To determine whether cellular expression of RAGE was necessary for resistance to RSV-induced cell death, we examined the effects of soluble RAGE. HEK cells were pre-treated with anti-RSV F protein (palivizumab) or RAGE-Fc (RAGE expressed as a fusion protein with human Fc), infected with RSV at an m.o.i. of 1 and the ATP levels were assessed after 48 h. In line with previous studies that have shown that anti-RSV F protein inhibits RSV infection and F-protein-mediated syncytium formation (Huang *et al.*, 2010b), anti-F protein efficiently protected the HEK cells from RSV-induced cell death (Fig. 2c). Interestingly, soluble RAGE-Fc also provided significant protection, blocking ~50 % of the reduction in ATP levels mediated by the RSV infection (Fig. 2c). These data indicated that cellular expression of RAGE is not necessarily required to inhibit RSV-induced cell death. Moreover, as membrane and soluble expression of RAGE failed to impede the initial infection, transcription and generation of the F protein (Fig. 1b, d), these data indicated that RAGE influences a later stage of viral infection.

**Fig. 3.**
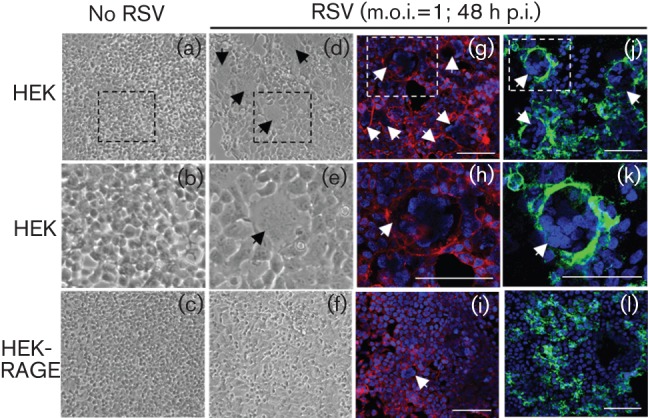
RAGE inhibits RSV-induced syncytium formation. HEK and HEK-RAGE cell cultures were infected with live RSV (m.o.i. = 1) for 48 h as indicated and photographed. Light micrograghs (a–f) were taken at 10× magnification and syncytia are indicated with black arrows; the boxed regions in (a) and (d) are enlarged in (b) and (e), respectively. Confocal images (g–l) were taken at 40× magnification and syncytia are indicated with white arrows. Cells were visualized with a membrane stain (red; g–i), Alexa Fluor 488-labelled anti-F protein antibody (green) (j–l) and Hoechst 34580 nuclei staining (blue) (g–l). The boxes in (g) and (j) are enlarged to show increased detail in (h) and (k), respectively. Bars, 100 µM (g–l).

A feature of severe lower respiratory infections with RSV is fusion of cells and the formation of multinucleate syncytia (which is also mediated by the F protein), and cell sloughing. Syncytia are also associated with increased viral titres and may enable the transfer of viral particles between neighbouring cells in a manner that limits exposure of the virus to the host immune system. To determine whether RAGE impacted the generation of syncytia, parental HEK and HEK-RAGE cells were infected with RSV and examined by light and confocal microscopy. In the first 24 h following RSV infection, no obvious syncytia were detectable, but by 48 h p.i. multinucleate syncytia were readily observed in the parental HEK cultures (Fig. 3). Using light microscopy, the syncytia were be identified by the large lesions with poor cellular definition (compare Fig. 3a–c with Fig. 3d, e). In contrast, such lesions were not apparent in the RSV-infected HEK-RAGE cultures (Fig. 3f). Confocal microscopy was used to confirm the cell fusion and multinucleate nature of the lesions and the protection afforded by the expression of RAGE using either cell membrane and nuclei staining (Fig. 3g–i) or F-protein and nuclei staining (Fig. 3j–l). By 72 h p.i., the HEK cultures largely lacked cellular integrity, and this was associated with substantial sloughing, which was consistent with the significant reductions in ATP levels and the reduced proportion of live cells recovered from cultures at this time point (Fig. 2a, b).

**Fig. 4.**
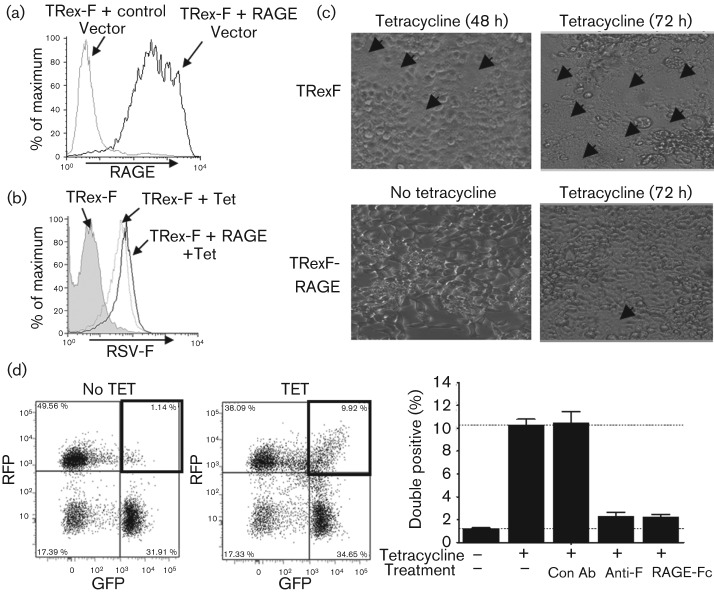
RAGE inhibits F-mediated syncytium formation in a virus-free system. RSV F-protein-mediated syncytium formation was demonstrated in a virus-free F-protein-expressing cell line, TRex-F. (a) TRex-F cells were transiently transfected with human RAGE or control vector and RAGE expression was confirmed by FACS. (b, c) RSV F-protein levels were determined by FACS 24 h after F protein was induced with tetracycline (b), and 48 and 72 h after induction of F protein, the occurrence of syncytia (indicated by arrows) was examined by light microscopy (c). (d) Transiently transfected RFP and GFP TRex-F cells were mixed (1 : 1 ratio) and incubated without or with tetracycline alone or in the presence of control antibody (Con Ab; 600 nM), anti-RSV F-protein antibody (60 nM) or RAGE-Fc (600 nM), and double-positive fusion events were examined after 48 h by FACS.

To demonstrate further that RAGE could interfere with F-protein mediated syncytium formation, we utilized a virus-free tetracycline-inducible F-protein-expressing cell line, TRex-F, which was developed previously to specifically examine syncytium formation (Huang *et al.*, 2010b). Full-length RAGE was transiently transfected into TRex-F cells and expression was confirmed by FACS (Fig. 4a). TRex-F cells expressed F protein as early as 3 h after tetracycline treatment. Expression of RAGE did not alter the levels of F protein (Fig. 4b). Consistent with previous data describing this model system (Huang *et al.*, 2010b), small syncytia were observed 48 h after the induction of F protein, and extensive syncytia were apparent by 72 h (Fig. 4c). In contrast, syncytia were virtually absent in RAGE-transfected TRex-F cells 72 h after F-protein induction (Fig. 4c). Quantification of the size and number of syncytia by microscopy is quite challenging, so we adopted a previously developed FACS-based approach to quantify early fusion events (Huang *et al.*, 2010b). F-protein expression was induced in a mixture of GFP and red fluorescent protein (RFP)-expressing TRex-F cells and the frequency of fusion events between red and green cells was determined. Two days after induction of F protein, double-positive fusion events represented ~10 % of the cells and these fusion events were inhibited by anti-RSV F-protein antibody or RAGE-Fc to background levels (Fig. 4d).

**Fig. 5.**
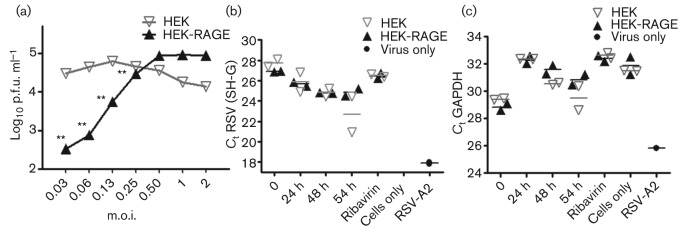
RAGE reduces viral titres but does not appear to impact virus replication or budding during the early stages of infection. (a) To assess viral propagation, 2.5×10^4^ HEK or HEK-RAGE cells were infected with RSV at the indicated m.o.i. After 2 h, cells were washed to remove the inoculum and at 72 h p.i. and the number of infectious particles recovered from culture supernatants was determined by plaque assay. Parametric *t*-tests were used to determine whether the viral titres between the HEK and HEK-RAGE cells differed significantly. **P<0.01. (b, c) RSV replication and the release of viral particles (budding) into the supernatants was determined with PCR assays. (b) Levels of genomic RSV SH-G were used to assess the levels of viral particles. Results indicated by ‘Cells only’ had an RSV SH–G *C*_t_ value of 45 for both HEK and HEK-RAGE. (c) To control for viral particles associated with sloughing cells into the supernatants, the levels of GAPDH were also determined. RSV SH–G and GAPDH levels are represented as the *C*_t_ values for HEK (grey inverted triangles) and HEK-RAGE (black triangles) cells assessed at the 0, 24, 48 and 54 h time points, along with ribavain (inhibitor of virus replication) and cells-only and virus-only controls.

As indicated previously, syncytia are apparent at ~48 h after infection, which generally precedes the cell sloughing and viral titres that are notable at 72 h. To determine whether the RAGE-dependent inhibition of syncytia was associated with a reduction in viral production, we examined the number of infectious particles recovered from the supernatants at 72 h p.i. Supernatants harvested from HEK cultures had significantly higher viral titres than HEK-RAGE cultures at all viral doses up to an m.o.i. of 0.25 when the viral titres had more or less plateaued (Fig. 5a). At higher viral doses, the titres declined for the HEK culture, although this was probably influenced by the substantial reduction in cell survival of the HEK cultures at these viral doses at 48 and 72 h p.i. (Fig. 2a, b).

So far, the data indicated that RAGE inhibits RSV-induced syncytium formation, cell death and associated viral release. The impact of soluble RAGE on cell survival and syncytium formation indicated that RAGE expression by the host cell is not necessary to facilitate its effects. However, it does not preclude the possibility that intrinsic expression of RAGE may also alter virus replication and budding. To investigate this possibility, the levels of viral genomes liberated from cultures were examined over time using an RSV SH–G gene PCR assay (Huang *et al.*, 2010b). A decrease in the cycle threshold (*C*_t_) over time corresponds to an increase in the viral genomes detected in the culture supernatants when compared with cells exposed to ribavirin, which inhibits virus replication. At the early 24 and 48 h p.i. time points, there was no apparent difference in the viral genomes released from the HEK and HEK-RAGE cultures (Fig. 5b). By 54 h p.i., there was a trend towards an increase in the amount of virus detected in the HEK supernatants, which was consistent with increased syncytia and cell sloughing and an increase in the mammalian glyceraldehyde 3-phosphate dehydrogenase (GAPDH) transcripts detected at these time points (Fig. 5c). These data indicated that RAGE does not appear to influence virus replication and budding during the early stages of infection; rather, RAGE appears to impact directly on syncytium formation, cell viability and cell sloughing, which in turn affect viral titres. However, it is plausible that expression of RAGE may also increase the tolerance of cells to RSV infection, and this may also influence cell survival, syncytium formation and cell sloughing.

**Fig. 6.**
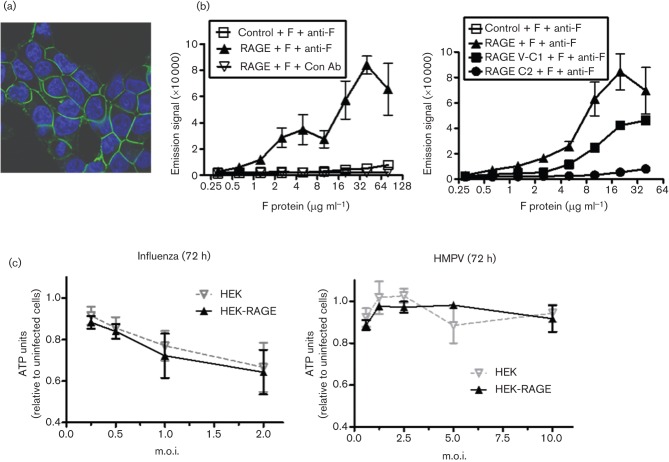
RAGE is associated with cell-to-cell contacts and interacts with RSV F protein but does not impact the survival of influenza virus or human metapneumovirus (HMPV)-infected cells. (a) Confocal microscopy of HEK-RAGE-CFP. (B) Binding of the extracellular region of human RAGE to RSV F protein was assessed by an AlphaScreen assay. Recombinant RSV F protein, histidine-tagged control protein, full-length extracellular RAGE (left panel) or truncated V-C1 (aa 1–258) or C2 (aa 234–342) domains of extracellular RAGE (right panel) and anti-RSV F-protein antibody (anti-F) were pre-incubated for 30 min and then nickel-labelled donor beads and protein A-labelled acceptor beads were added for 10 min, and excitation was measured on a Wallac EnVision instrument. (c) HEK (grey inverted triangles) and HEK-RAGE (black triangles) cells were seeded and infected with influenza virus or HMPV, and ATP levels were measured after 72 h. Units are expressed relative to uninfected cell cultures.

As soluble and membrane-expressed forms of RAGE could impede the formation of syncytia, we investigated the possibility that RAGE directly interacts with RSV F protein. Confocal examination HEK-RAGE-CFP (cyan fluorescent protein) cells demonstrated that RAGE is expressed predominantly at the contact points between cells (Fig. 6a), which is consistent with its capacity to block F protein-mediated syncytium formation. Moreover, using an AlphaScreen assay, we demonstrated that full-length extracellular RAGE, which includes the V/C1 and C2 domains, could interact directly with recombinant RSV F protein (Fig. 6b). The high concentrations of F protein necessary to generate a signal indicated that this is a low affinity interaction, but the remarkably high expression levels of RAGE on the lower airway epithelial cells would suggest that it may be a physiologically relevant interaction. Using truncated variants of the RAGE extracellular region, we determined that the binding was mediated primarily by the V/C1 domains (Fig. 6b). This region is crucial for binding a diverse range of ligands, and the interactions involve a combination of structural motifs and exposed positively charged residues (Park *et al.*, 2010). Further experimentation would be necessary to confirm and detail the fine specificities between RSV F protein and RAGE, and whether these findings extend to closely related viruses. However, preliminary data indicated that RAGE expression had no significant effect on the viability of HEK293 cells infected with two other enveloped respiratory viruses, influenza and human metapneumovirus (HMPV) (Fig. 6c), suggesting that, at least in this case, RAGE may not be able to interfere with the consequences of influenza or HMPV infection.

Together, these *in vitro* data indicated that soluble and membrane-expressed RAGE promote the survival of RSV-infected cells and inhibit syncytia and cell sloughing, and probably as a consequence reduce viral titres, and these effects may be mediated by a direct interaction between RAGE and RSV F protein. Rather than blocking the initial interaction and entry of viral particles into target cells, evidence from both viral infection and virus-free inducible F-protein systems indicate that RAGE interferes with F-protein-mediated cell-to-cell fusion and the formation of syncytia. Interestingly, RSV F protein is important for both viral attachment and cell-to-cell fusion, but RAGE only appears to be able to impact the later. It is not clear why this is the case. Biochemical data indicate that RAGE can directly interact with the F protein, so it was conceivable that RAGE, like anti-F protein antibody, may influence both processes. Fine mapping of the interactions may provide further insight. Previous characterization of type I lung epithelial cells indicates that RAGE appears to be expressed predominantly on the basal membrane and where membranes juxtapose rather than on the apical membrane (Shirasawa *et al.*, 2004). Confocal microscopy of HEK-RAGE cells also shows a similar membrane distribution so it is plausible that RAGE would fail to inhibit infection of apical membranes but block cell-to-cell fusion. However, this explanation seems unlikely, as soluble RAGE was able to block cell-to-cell fusion but was also unable to impact on initial infection. The factors that differentially influence these F-protein-mediated functions are largely unknown. RAGE appears to specifically impact F-protein-mediated syncytium formation.

It remains uncertain whether RAGE inhibition of syncytium formation is the sole element responsible for the reduction in RSV titres and the survival advantage of infected cells. It is plausible that expression of RAGE may independently promote the survival of virally infected cells in addition to blocking syncytium formation, or these events could be intimately linked. It could be similarly argued that RAGE could inhibit the generation of viral particles independently of the blockade of syncytia; however, prior to the formation of syncytia, the generation and release of viral particles does not appear to be influenced by RAGE. It therefore appears that prevention of cell-to-cell fusion and subsequent cellular sloughing may be the critical aspect. Nevertheless, the mechanism by which RAGE promotes survival and inhibits syncytium formation warrants further investigation.

A recent study demonstrated that RSV causes syncytium formation, apical sloughing and apoptosis of well-differentiated paediatric bronchial epithelial cultures independently of an immune response (Villenave *et al.*, 2012). Studies examining the expression and RAGE and the impact of soluble RAGE on differentiated cell cultures from relevant clinical samples infected with RSV would be of significant value. The different effects of RSV infections on well-differentiated bronchial epithelial cultures derived from adults would suggest that factors probably associated with susceptible groups impact on the pathology and disease severity *in vitro* and *in vivo*. RAGE may be one such factor. It is certainly conceivable that differential RAGE expression in the lung may influence the sensitivity to a more severe RSV infection. There is indeed a precedent for differential RAGE expression in the terminal airways in the rat. RAGE is first detected during late embryonic development and expression gradually increases until after birth (Shirasawa *et al.*, 2004). Examination of the expression levels in infant and adult non-human primates would be informative, and assessment of RAGE expression in lambs would also be useful, as premature lambs are known to develop a more severe RSV infection with a greater prevalence of syncytia and increased tissue pathology compared with neonates (Meyerholz *et al.*, 2004).

RAGE is expressed at very high levels by type I pneumocystis, and soluble RAGE liberated by RSV infection can scavenge inflammatory mediators and perturb host inflammatory responses (Buckley & Ehrhardt, 2010; Miller *et al.*, 2012). The data presented here indicate that RAGE may also independently promote the survival of RSV-infected cells and inhibit the formation of syncytia and cell sloughing and limit viral spread. The combination of these mechanisms may provide protection from the potentially catastrophic consequences of severe lower respiratory tract RSV infection.

## Methods

### 

#### Reagents.

Full-length RAGE (GenBank accession no. NM_001136) was cloned into pcDNA3.1 and stably transfected into HEK293 cells. Cells were grown initially in antibiotic selection medium and sorted by FACS. HEK and HEK-RAGE cells were otherwise maintained without selection. Expression of cell-surface RAGE was checked periodically with anti-RAGE antibody clone 4F4 (MedImmune LLC) by flow cytometry. HEK293 cells stably transfected with full-length RAGE-CFP was a kind gift from Eicke Latz (University of Bonn). The generation of anti-RSV F-protein antibody (Medi-493, palivizumab) and of RSV F protein has been described previously (Johnson *et al.*, 1997; Wu *et al.*, 2007).

#### Cell-survival assays.

Parental HEK or HEK-RAGE cells were seeded at 1×10^4^ cells in a 96-well culture plate and incubated alone or with an increasing m.o.i. of virus. After 24, 48 and 72 h, cellular ATP levels were measured as an indirect assessment of cell-culture viability using a CellTiter-Glo assay (Promega). ATP units were expressed relative to uninfected cell cultures. In some experiments, cell cultures were treated with 300 nM soluble RAGE-Fc, 30 nM anti-RSV F protein (palivizumab) or 300 nM control IgG. Live cell counts were determined directly using a Vi-Cell XR (Beckman Coulter) using trypan blue without or with RSV infection (m.o.i. of 1) after 24, 48 and 72 h. Live cell counts were expressed relative to uninfected cells.

#### RSV A2 virus propagation.

RSV A2 virus was cultivated in Hep2 cells. Cells were infected with virus at an m.o.i. of 0.1 in medium with 10 % FBS, and at 4–5 days p.i., viable virion particles were harvested from infected cultures by performing multiple freeze–thaw cycles on the infected cell pellet. The suspension was clarified by centrifugation and the supernatant containing infectious virions was stored at −80 °C. Titres were determined by plaque assay on Hep2 cells.

#### Influenza virus propagation.

Viable 9–11-day-old embryonated eggs were infected with 100 µl 1 : 10 000-diluted influenza virus strain A/PR8/34 VR-95 D3 1000 viral stock and incubated for 72 h at 35 °C in an egg incubator. The eggs were then chilled at 4 °C overnight. The allantoic fluid containing virus was harvested, the haemagglutination titre was determined and 1 ml aliquots were frozen on dry ice and stored at −70 °C.

#### HMPV virus propagation.

Vero cells were infected with HMPV 001 at an m.o.i. of 0.1 in OPTI-MEM medium and incubated at 37 °C for 2 h. The viral inoculum was then removed and the cell layer was covered with fresh OPTI-MEM medium, incubated at 37 °C and monitored daily by microscopy. The viral supernatants were harvested when ~60 % of the cells were dead/floating. Following a single freeze–thaw cycle, cellular debris was pelleted by centrifugation and viral supernatants were collected. The virus was stabilized in 1× SPG (74.62 g sucrose l^−1^, 0.517 g KH_2_PO_4_ l^−1^, 1.254 g K_2_HPO_4_ l^−1^, 1.01 g l-glutamic acid monosodium salt l^−1^) and stored in aliquots at −80 °C. The virus stock titre was determined by an F-protein-expression ELISA.

#### AlphaScreen assay.

Binding of the extracellular region of human RAGE to RSV F protein was assessed using an AlphaScreen assay (Perkin Elmer). Histidine-tagged control protein, full-length extracellular RAGE or truncated variants of the RAGE extracellular domain, V-C1 (aa 1–258) or C2 (a 234–342) (all 100 nM), anti-RSV F-protein antibody (25 nM) and range of concentrations of recombinant RSV F protein were pre-incubated for 30 min. Nickel-labelled donor beads and protein A-labelled acceptor beads were then added for 10 min, and excitation was measured on a Wallac EnVision (Perkin Elmer) instrument.

#### Light and confocal microscopy.

HEK and HEK-RAGE cells were seeded at 5×10^4^ cells per well (24-well culture plates) for light microscopy or 1×10^5^ poly-l-lysine-coated eight-well chamber slides for confocal microscopy, and infected with live RSV (m.o.i. = 1) and cultured for 48 or 72 h. Light micrographs were taken with a 10× objective. For confocal microscopy, RSV-infected cells were stained with Alexa Fluor 488-labelled anti-F antibody (0.5 µg ml^−1^; palivizumab) and Hoechst 34580, or with CellMask Deep Red plasma membrane stain (Life Technologies) and Hoechst 34580. Images were captured on a Leica SP5 confocal microscope using a 40× objective.

#### Virus-free syncytia assays.

Virus-free syncytia assays using tetracycline-inducible TRex-F cells for qualitative and quantitative assessment of syncytia have been described previously (Huang *et al.*, 2010b). TRex-F cells were transiently transfected with human RAGE or control vector. At 24 h after transfection, tetracycline was added to induce the expression of F protein on the cell membrane. The formation of syncytia was assessed after 48 and 72 h by microscopy. To quantitatively assess syncytia, transiently transfected RFP and GFP TRex-F cells were mixed (1 : 1 ratio) and allowed to settle overnight, and then incubated without or with tetracycline alone or in the presence of control antibody (600 nM), anti-RSV F antibody (60 nM) or RAGE-Fc (600 nM), and double-positive fusion events were examined after 48 h by FACS.

#### Budding assay.

Parental HEK and HEK-RAGE cells were infected at an m.o.i. of 1 with RSV A2 virus for 1 h and supernatants were replaced with fresh medium to remove unbound virus. Cells were then incubated for 24, 48 and 54 h and the supernatants were harvested. Ribavirin (100 µM), an inhibitor of RSV replication, and cell-only controls were also included and were harvested at 72 h. RNA extracts were prepared from 140 µl cell-culture supernatant using a QIAamp Viral RNA Mini kit (Qiagen). One-step quantitative reverse transcription-PCR was performed by using an AgPath ID One-Step RT-PCR kit (Applied Biosystems). RSV A2 primers covering a conserved region over the SH and G genes were used to amplify the viral genome and antigenome and GAPDH primers to amplify the mammalian housekeeping gene. The primers, FAM reporter probe and VIC reporter probe mix were obtained from Applied Biosystems. Amplification was performed on a 7900HT FAST RT-PCR system (Applied Biosystems) using the following thermocycling conditions: 30 min at 48 °C for reverse transcription, 10 min at 95 °C for polymerase activation, and then 45 cycles of 15 s at 95 °C and 1 min at 60 °C. Fluorescence was read at the combined annealing/extension step at 60 °C and recorded as *C*_t_ values.

## References

[r1] Bem R. A. Domachowske J. B. Rosenberg H. F. **(**2011**).** Animal models of human respiratory syncytial virus disease. Am J Physiol Lung Cell Mol Physiol 301, L148–L156 10.1152/ajplung.00065.2011 21571908 PMC3154630

[r2] Buckley S. T. Ehrhardt C. **(**2010**).** The receptor for advanced glycation end products (RAGE) and the lung. J Biomed Biotechnol 2010, 917108 10.1155/2010/917108 20145712 PMC2817378

[r3] Collins P. L. Melero J. A. **(**2011**).** Progress in understanding and controlling respiratory syncytial virus: still crazy after all these years. Virus Res 162, 80–99 10.1016/j.virusres.2011.09.020 21963675 PMC3221877

[r4] Ferhani N. Letuve S. Kozhich A. Thibaudeau O. Grandsaigne M. Maret M. Dombret M.-C. Sims G. P. Kolbeck R. **& other authors (**2010**).** Expression of high-mobility group box 1 and of receptor for advanced glycation end products in chronic obstructive pulmonary disease. Am J Respir Crit Care Med 181, 917–927 10.1164/rccm.200903-0340OC 20133931

[r5] Hallak L. K. Spillmann D. Collins P. L. Peeples M. E. **(**2000**).** Glycosaminoglycan sulfation requirements for respiratory syncytial virus infection. J Virol 74, 10508–10513 10.1128/JVI.74.22.10508-10513.2000 11044095 PMC110925

[r7] Huang K. Lawlor H. Tang R. MacGill R. S. Ulbrandt N. D. Wu H. **(**2010a**).** Recombinant respiratory syncytial virus F protein expression is hindered by inefficient nuclear export and mRNA processing. Virus Genes 40, 212–221 10.1007/s11262-010-0449-8 20111897

[r8] Huang K. Incognito L. Cheng X. Ulbrandt N. D. Wu H. **(**2010b**).** Respiratory syncytial virus-neutralizing monoclonal antibodies motavizumab and palivizumab inhibit fusion. J Virol 84, 8132–8140 10.1128/JVI.02699-09 20519399 PMC2916538

[r9] Johnson S. Oliver C. Prince G. A. Hemming V. G. Pfarr D. S. Wang S. C. Dormitzer M. O’Grady J. Koenig S. **& other authors (**1997**).** Development of a humanized monoclonal antibody (MEDI-493) with potent in vitro and in vivo activity against respiratory syncytial virus. J Infect Dis 176, 1215–1224 10.1086/514115 9359721

[r10] Johnson J. E. Gonzales R. A. Olson S. J. Wright P. F. Graham B. S. **(**2007**).** The histopathology of fatal untreated human respiratory syncytial virus infection. Mod Pathol 20, 108–119 10.1038/modpathol.3800725 17143259

[r11] Kahn J. S. Schnell M. J. Buonocore L. Rose J. K. **(**1999**).** Recombinant vesicular stomatitis virus expressing respiratory syncytial virus (RSV) glycoproteins: RSV fusion protein can mediate infection and cell fusion. Virology 254, 81–91 10.1006/viro.1998.9535 9927576

[r12] König P. Giesow K. Schuldt K. Buchholz U. J. Keil G. M. **(**2004**).** A novel protein expression strategy using recombinant bovine respiratory syncytial virus (BRSV): modifications of the peptide sequence between the two furin cleavage sites of the BRSV fusion protein yield secreted proteins, but affect processing and function of the BRSV fusion protein. J Gen Virol 85, 1815–1824 10.1099/vir.0.80010-0 15218165

[r13] Leyssen P. De Clercq E. Neyts J. **(**2008**).** Molecular strategies to inhibit the replication of RNA viruses. Antiviral Res 78, 9–25 10.1016/j.antiviral.2008.01.004 18313769 PMC7114363

[r14] Meissner H. C. Rennels M. B. Pickering L. K. Hall C. B. **(**2004**).** Risk of severe respiratory syncytial virus disease, identification of high risk infants and recommendations for prophylaxis with palivizumab. Pediatr Infect Dis J 23, 284–285 10.1097/01.inf.0000121203.33560.f9 15014320

[r15] Melikyan G. B. Markosyan R. M. Hemmati H. Delmedico M. K. Lambert D. M. Cohen F. S. **(**2000**).** Evidence that the transition of HIV-1 gp41 into a six-helix bundle, not the bundle configuration, induces membrane fusion. J Cell Biol 151, 413–424 10.1083/jcb.151.2.413 11038187 PMC2192659

[r16] Meyerholz D. K. Grubor B. Fach S. J. Sacco R. E. Lehmkuhl H. D. Gallup J. M. Ackermann M. R. **(**2004**).** Reduced clearance of respiratory syncytial virus infection in a preterm lamb model. Microbes Infect 6, 1312–1319 10.1016/j.micinf.2004.08.006 15555538 PMC2791065

[r17] Miller A. L. Sims G. P. Brewah Y. A. Rebelatto M. C. Kearley J. Benjamin E. Keller A. E. Brohawn P. Herbst R. **& other authors (**2012**).** Opposing roles of membrane and soluble forms of receptor for advanced glycation endproducts (RAGE) in primary respiratory syncytial virus infection. J Infect Dis 205, 1311–1320 10.1093/infdis/jir826 22262795 PMC3308901

[r18] Morton C. J. Cameron R. Lawrence L. J. Lin B. Lowe M. Luttick A. Mason A. McKimm-Breschkin J. Parker M. W. **& other authors (**2003**).** Structural characterization of respiratory syncytial virus fusion inhibitor escape mutants: homology model of the F protein and a syncytium formation assay. Virology 311, 275–288 10.1016/S0042-6822(03)00115-6 12842618

[r19] Neilson K. A. Yunis E. J. **(**1990**).** Demonstration of respiratory syncytial virus in an autopsy series. Pediatr Pathol 10, 491–502 10.3109/15513819009067138 1695371

[r20] Olivier A. Gallup J. de Macedo M. M. Varga S. M. Ackermann M. **(**2009**).** Human respiratory syncytial virus A2 strain replicates and induces innate immune responses by respiratory epithelia of neonatal lambs. Int J Exp Pathol 90, 431–438 10.1111/j.1365-2613.2009.00643.x 19659901 PMC2741153

[r21] Papin J. F. Wolf R. F. Kosanke S. D. Jenkins J. D. Moore S. N. Anderson M. P. Welliver R. C. Sr **(**2013**).** Infant baboons infected with respiratory syncytial virus develop clinical and pathological changes that parallel those of human infants. Am J Physiol Lung Cell Mol Physiol 304, L530–L539 10.1152/ajplung.00173.2012 23418091 PMC3625990

[r22] Park H. Adsit F. G. Boyington J. C. **(**2010**).** The 1.5 Å crystal structure of human receptor for advanced glycation endproducts (RAGE) ectodomains reveals unique features determining ligand binding. J Biol Chem 285, 40762–40770 10.1074/jbc.M110.169276 20943659 PMC3003376

[r23] Raucci A. Cugusi S. Antonelli A. Barabino S. M. Monti L. Bierhaus A. Reiss K. Saftig P. Bianchi M. E. **(**2008**).** A soluble form of the receptor for advanced glycation endproducts (RAGE) is produced by proteolytic cleavage of the membrane-bound form by the sheddase a disintegrin and metalloprotease 10 (ADAM10). FASEB J 22, 3716–3727 10.1096/fj.08-109033 18603587

[r24] Russell C. J. Jardetzky T. S. Lamb R. A. **(**2001**).** Membrane fusion machines of paramyxoviruses: capture of intermediates of fusion. EMBO J 20, 4024–4034 10.1093/emboj/20.15.4024 11483506 PMC149161

[r25] Shirasawa M. Fujiwara N. Hirabayashi S. Ohno H. Iida J. Makita K. Hata Y. **(**2004**).** Receptor for advanced glycation end-products is a marker of type I lung alveolar cells. Genes Cells 9, 165–174 10.1111/j.1356-9597.2004.00712.x 15009093

[r26] Sidwell R. W. Barnard D. L. **(**2006**).** Respiratory syncytial virus infections: recent prospects for control. Antiviral Res 71, 379–390 10.1016/j.antiviral.2006.05.014 16806515

[r27] Sims G. P. Rowe D. C. Rietdijk S. T. Herbst R. Coyle A. J. **(**2010**).** HMGB1 and RAGE in inflammation and cancer. Annu Rev Immunol 28, 367–388 10.1146/annurev.immunol.021908.132603 20192808

[r28] Sow F. B. Gallup J. M. Olivier A. Krishnan S. Patera A. C. Suzich J. Ackermann M. R. **(**2011**).** Respiratory syncytial virus is associated with an inflammatory response in lungs and architectural remodeling of lung-draining lymph nodes of newborn lambs. Am J Physiol Lung Cell Mol Physiol 300, L12–L24 10.1152/ajplung.00169.2010 20935230 PMC3023288

[r29] Tayyari F. Marchant D. Moraes T. J. Duan W. Mastrangelo P. Hegele R. G. **(**2011**).** Identification of nucleolin as a cellular receptor for human respiratory syncytial virus. Nat Med 17, 1132–1135 10.1038/nm.2444 21841784

[r30] Techaarpornkul S. Barretto N. Peeples M. E. **(**2001**).** Functional analysis of recombinant respiratory syncytial virus deletion mutants lacking the small hydrophobic and/or attachment glycoprotein gene. J Virol 75, 6825–6834 10.1128/JVI.75.15.6825-6834.2001 11435561 PMC114409

[r31] Tristram D. A. Hicks W. Jr Hard R. **(**1998**).** Respiratory syncytial virus and human bronchial epithelium. Arch Otolaryngol Head Neck Surg 124, 777–783 10.1001/archotol.124.7.777 9677113

[r32] Uchida T. Shirasawa M. Ware L. B. Kojima K. Hata Y. Makita K. Mednick G. Matthay Z. A. Matthay M. A. **(**2006**).** Receptor for advanced glycation end-products is a marker of type I cell injury in acute lung injury. Am J Respir Crit Care Med 173, 1008–1015 10.1164/rccm.200509-1477OC 16456142 PMC2662912

[r33] Villenave R. Thavagnanam S. Sarlang S. Parker J. Douglas I. Skibinski G. Heaney L. G. McKaigue J. P. Coyle P. V. **& other authors (**2012**).** In vitro modeling of respiratory syncytial virus infection of pediatric bronchial epithelium, the primary target of infection in vivo. Proc Natl Acad Sci U S A 109, 5040–5045 10.1073/pnas.1110203109 22411804 PMC3323997

[r34] Wu H. Pfarr D. S. Johnson S. Brewah Y. A. Woods R. M. Patel N. K. White W. I. Young J. F. Kiener P. A. **(**2007**).** Development of motavizumab, an ultra-potent antibody for the prevention of respiratory syncytial virus infection in the upper and lower respiratory tract. J Mol Biol 368, 652–665 10.1016/j.jmb.2007.02.024 17362988

[r35] Yamakawa N. Uchida T. Matthay M. A. Makita K. **(**2011**).** Proteolytic release of the receptor for advanced glycation end products from in vitro and in situ alveolar epithelial cells. Am J Physiol Lung Cell Mol Physiol 300, L516–L525 10.1152/ajplung.00118.2010 21257730 PMC3075094

[r36] Yin H. S. Paterson R. G. Wen X. Lamb R. A. Jardetzky T. S. **(**2005**).** Structure of the uncleaved ectodomain of the paramyxovirus (hPIV3) fusion protein. Proc Natl Acad Sci U S A 102, 9288–9293 10.1073/pnas.0503989102 15964978 PMC1151655

[r37] Yin H. S. Wen X. Paterson R. G. Lamb R. A. Jardetzky T. S. **(**2006**).** Structure of the parainfluenza virus 5 F protein in its metastable, prefusion conformation. Nature 439, 38–44 10.1038/nature04322 16397490 PMC7095149

[r38] Zhang L. Peeples M. E. Boucher R. C. Collins P. L. Pickles R. J. **(**2002**).** Respiratory syncytial virus infection of human airway epithelial cells is polarized, specific to ciliated cells, and without obvious cytopathology. J Virol 76, 5654–5666 10.1128/JVI.76.11.5654-5666.2002 11991994 PMC137037

